# Case report: Intra-abdominal aggressive fibromatosis: A rare cause of hyperemesis

**DOI:** 10.3389/fsurg.2023.1108225

**Published:** 2023-02-21

**Authors:** Zilin Zou, Guannan Ye, Saiqun Xu, Wei Liu, Weining Wang

**Affiliations:** ^1^ The Affiliated Changsha Hospital, Hengyang Medical School, University of South China, Changsha, China; ^2^ Department of Gastroenterology, The First Hospital of Changsha, Changsha, China

**Keywords:** case report, aggressive fibromatosis (AF), desmoid-type fibromatosis, hyperemesis, APC

## Abstract

**Rationale:**

Aggressive fibromatosis is a rare and locally infiltrative monoclonal fibroblastic proliferation with lack of metastatic potential. We describe a rare case of intra-abdominal aggressive fibromatosis on young female with hyperemesis.

**Patient concerns:**

A 23-year-old female was admitted with hyperemesis and loss of weight.

**Diagnoses:**

According to imaging findings and immunohistology findings, a diagnosis of intra-abdominal aggressive fibromatosis was formulated.

**Outcomes:**

After the surgery, no evidence of local recurrence was noted during the 6 months of follow-up.

**Lessons:**

AF may explain why pregnant women may have severe hyperemesis.

## Introduction

Aggressive fibromatosis (AF), also known as desmoid-type fibromatosis (DT), is rare and tends to exhibit locally infiltrative monoclonal fibroblastic proliferation arising from connective tissues, and it can be found in any anatomical location with a lack of metastatic potential. AF can be divided into the following three types according to the location of the disease: abdominal AF, extra-abdominal AF and intra-abdominal AF. A young female patient with severe vomiting as the initial symptom was admitted to our hospital. The following report provides information for the diagnosis and treatment of such patients.

## Case report

**Figure 1:**
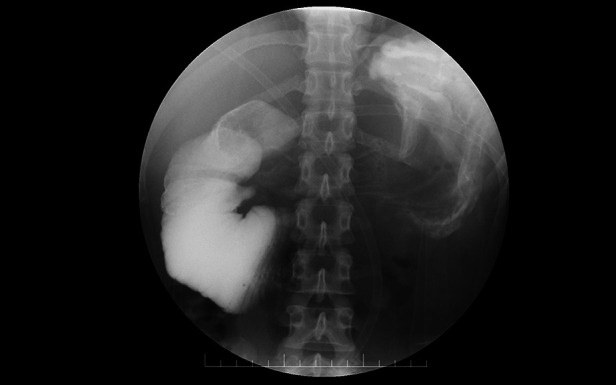
Gastrointestinal imaging shows duodenal stasis.

**Figure 2:**
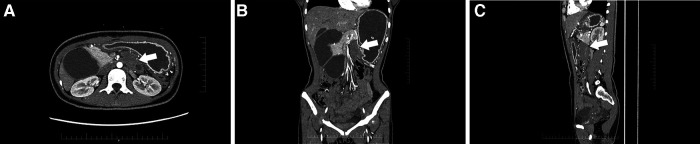
CT revealed a soft-tissue density mass about 5.4 cm*3.2 cm*5.8 cm (arrow), showing inhomogeneous density with ill-defined margin, and having a non-enhancing necrotic area with poor demarcation from the surrounding intestine (**A**) axial view, (**B**) coronal view, (**C**) sagittal view.

**Figure 3:**
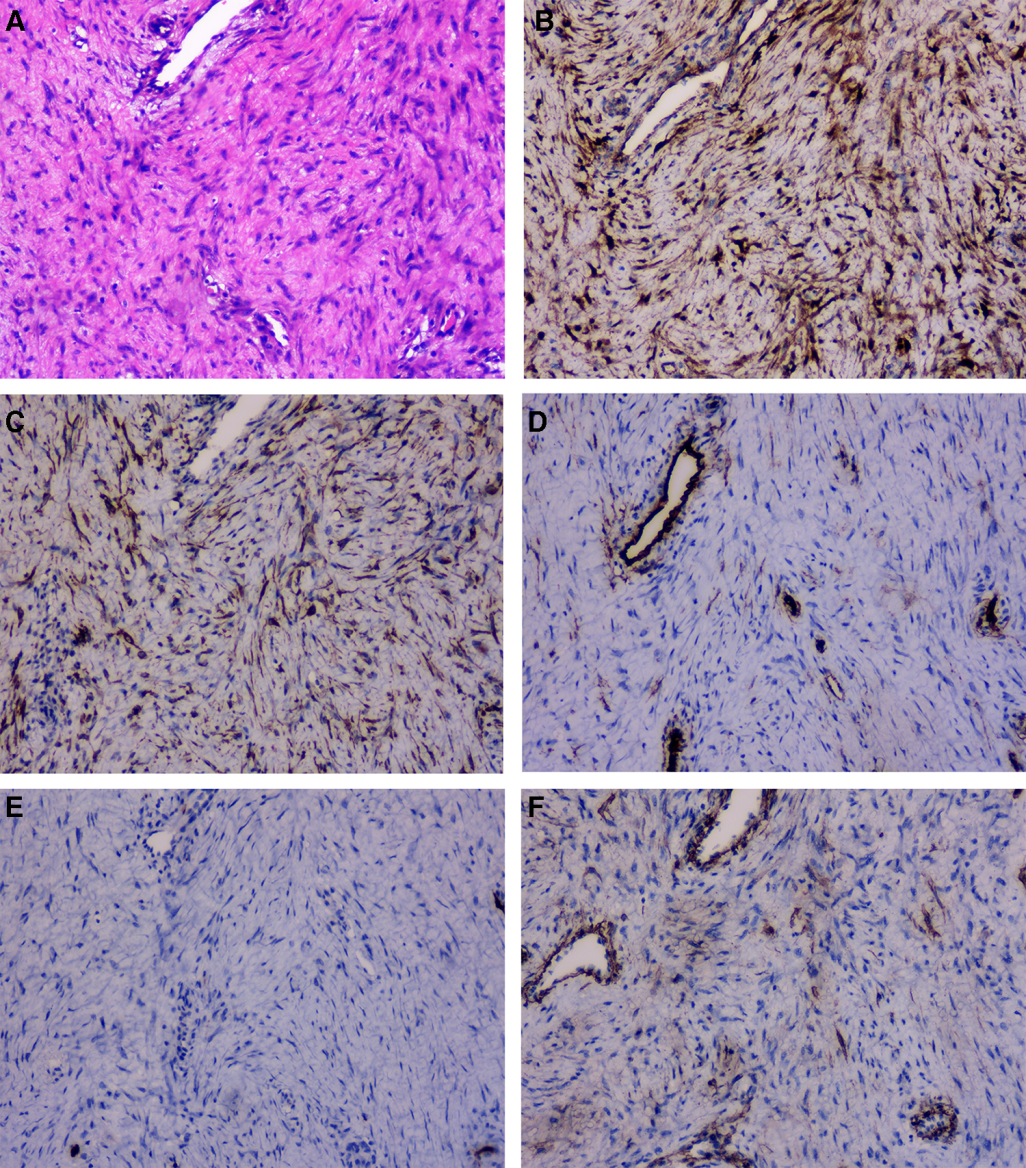
The tumor was composed of spindle cells (**A**, HE X100). Immunohistochemistry showed the spindle cells were positive for β-catenin (**B**, X100) and Desmin (**C**, X100), negative for CD34 (**D**, X100), S-100 (**E**, X100), SMA (**F**, X100).

The patient, a 23-year-old female, was admitted with hyperemesis and loss of weight. She experienced severe vomiting 30–60 min after a meal with no causes in March 2021 during the sixth month of gestation, and the vomiting continued until 2 months post-partum. In November 2021, the patient presented with vomiting after eating. The patient lost a total of 25 kg in the last 10 months. She was previously healthy, and she had no family history of these symptoms and signs. Upon the physical examination, succussion splashes were heard. Gastrointestinal imaging revealed a duodenal stasis (Figure 1). Enhanced computed tomography revealed a soft-tissue density mass about 5.4 cm*3.2 cm*5.8 cm (Figure 2, arrow), showing inhomogeneous density with ill-defined margin, and having a non-enhancing necrotic area with poor demarcation from the surrounding intestine. Due to the possible radiation injury, the patient refused to take the further image examinations. The patient didn't take the drugs because of her breastfeeding. An operation was performed on February 15, 2022, and a retroperitoneal mass was found on the left side of the mesenteric vessel, involving the posterior wall of the stomach, the body and the tail of pancreas, the spleen, and the splenic flexure. The patient underwent partial gastrectomy with gastrojejunostomy, total splenectomy, pancreatic body resection, pancreatic tail resection, partial colon resection, duodenal jejunum anastomosis and transverse colon-transverse colon anastomosis under general anaesthesia. Microscopic analysis identified that the tumour was composed of spindle-shaped fibroblasts (Figure 3A), and pathological diagnosis indicated aggressive fibromatosis. The following immunohistochemical results were observed: CD34 (−), CD117 (−), Desmin (+), DOG-1 (−), Actin (−), Ki67 (+5%), H-cald (−), STAT6 (plasma +), S100 (−), SMA (−), VEGF (−), β-catenin (+), ALK (−), CD68 (−), Vimentin (+), CKpan (−), ER (+10%), PR (+30%) (Figures 3B–F). Gene testing showed an APC mutation (p.S2552Cfs*29). The wound healed well after the operation, and clinical symptoms were not observed. According to these tests, a diagnosis of intra-abdominal aggressive fibromatosis was formulated. After the surgery, No evidence of local recurrence was noticed after the surgery and she had been gaining weight and been in good condition during the 6 months of follow-up.

## Discussion

Aggressive fibromatosis is a rare mesenchymal tumour with a high rate of local recurrence as well as a lack of capacity to metastasize (1), and AF has an overall good prognosis in terms of life expectancy. The incidence of aggressive fibromatosis has increased from 2.10 to 5.36 per million people per year. The median age of patients with AF is 39 years, and there is a trend of increasing age (2). AF can arise at any anatomical location of the body in different types of connective tissues. Due to the rare distant metastasis rate, the clinical symptoms are often apparent only after severe local invasion. Intra-abdominal AF accounts for approximately 10% of all AF cases (3), and its manifestations are commonly associated with abdominal pain and gastrointestinal bleeding (4).

The pathogenesis of AF is unclear and may be related to genetics, trauma, surgery, pregnancy or hormone levels. AF is closely associated with familial adenomatous polyposis (FAP). The risk of developing AF in patients with FAP is approximately 10%–15%, and AF associated with FAP is more aggressive (3). AF can occur after operations, such as laparoscopic and neck surgery, as well as after trauma (5). Surgical trauma is a causative factor that increases the production of β-catenin and promotes tumour development (6). The male–female ratio of AF patients shows an increasing female predominance, ranging from 68.6% in 1993–1998 to 73.6% in 2009–2013, which supports the influence of hormone levels in the development of AF (2). In sporadic cases, pregnancy is the most important influencing factor as it may result in abdominal trauma caused by oestrogen and uterine growth, thereby predisposing the patient to AF (7).

Aggressive fibromatosis is related to alterations in the Wnt/APC/β-catenin pathway. Mutations in the CTNNB1 gene, which is associated with β-catenin expression, are present in 70%–75% of AF cases, and the mutations often occur in exon 3. Among them, AF tumours carrying the S45F or S45P mutation in exon 3 are more often located in the abdominal wall than those carrying the T41A or T41I mutation and they have larger tumour sizes in cases with positive expression compared to patients with wild-type status in the CTNNB1 gene (8). Mutations in APC also occur in AF. CTNNB1 and APC mutations tend not to appear simultaneously. In contrast, patients with wild-type CTNNB1 should consider FAP and receive additional examinations to exclude FAP (9).

The treatment strategy for AF has changed. Due to its high postoperative recurrence rate, nonsurgical treatment for AF is recommended. There are two main treatment strategies for AF as follows: (1) active treatment and (2) frequent surveillance [also named the “wait-and-see” (W&S) approach].

For active treatment, systemic therapy is currently advocated as the main treatment. The main drugs include nonsteroidal anti-inflammatory drugs (NSAIDs), tyrosine kinase inhibitors (TKIs), gamma secretase inhibitors and Wnt pathway inhibitors. If prompt tumour shrinkage is important, it is necessary to select an anthracycline-based regimen because a tumour response may be achieved earlier (3). Although there is a preference in terms of the response rate with tyrosine kinase inhibitors, antiangiogenics are not the best option in acute abdominal presentations due to the risks of bleeding and suboptimal tissue repair (3). New targeted therapeutic agents, such as gamma secretase inhibitors and Wnt pathway inhibitors, have also emerged as potential options for future AF treatment, but more clinical studies are needed to support their efficacy (10). Because there is a low risk of local recurrence and a high benefit of surgery for abdominal wall AF, surgery is still a choice for abdominal wall AF. However, surgery is currently not a front-line or second-line treatment option. For symptomatic or growing tumours located at critical sites, such as the head and neck and scapular girdle, moderate-dose definitive radiotherapy has been employed as an alternative to surgery. Radiotherapy may also be considered in patients without significant drug efficacy (9). Currently, surgery combined with radiotherapy is not advocated in the primary treatment of AF; however, radiotherapy can be considered for the treatment of AF recurrence after surgery. High-intensity focused ultrasound is also an available option (11).

Shrinkage has been observed in 20% of intra-abdominal AF cases without any treatment (3). And the results have shown no differences in event-free survival and long-term disease control between patients who underwent surgery and those who underwent conservative treatment. Therefore, dynamic observation can also be performed under closely monitored conditions in patients without clinical symptoms, and if the patient does not progress within 2 years, any further aggressive treatment may not be required (9).

In our case, the patient was treated with surgery due to clinical symptoms, such as severe vomiting and weight loss, and the next step should be regular follow-up with close monitoring and radiotherapy if necessary. Women during pregnancy have an increased risk for the development of AF, and hyperemesis gravidarum is a common symptom for females who are pregnant. Thus, AF may explain why pregnant women may have severe hyperemesis.

## Data Availability

The datasets pertaining to this study can be found here: https://www.jianguoyun.com/p/DbzewJoQ_7uyCxjRtfUEIAA.
