# Supplementary figures and images for: Case report: Intra-abdominal aggressive fibromatosis: A rare cause of hyperemesis
Source: Front Surg. 2023 Feb 21;10:1108225. doi: 10.3389/fsurg.2023.1108225 (PMC9989300; doi:10.3389/fsurg.2023.1108225)

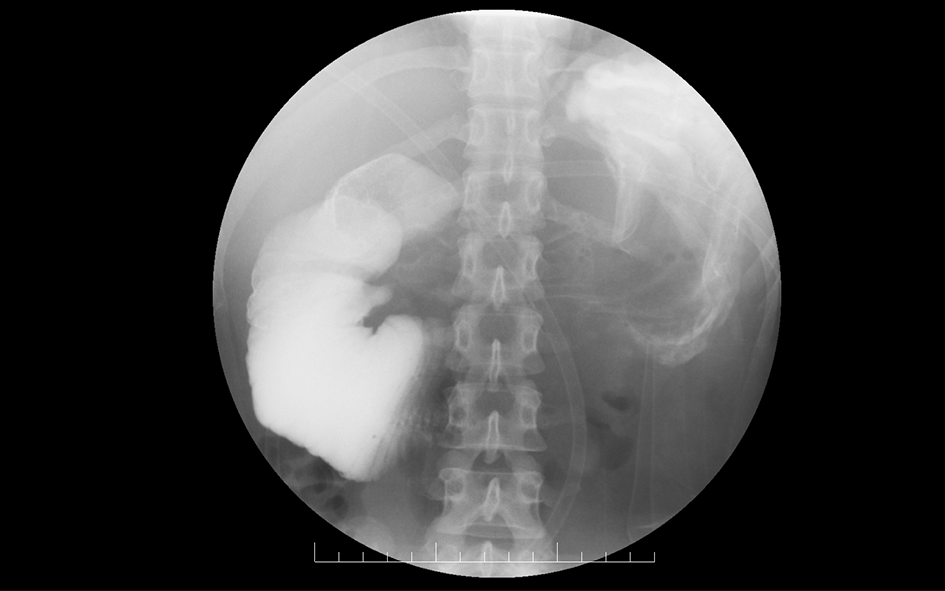

Supplement: Supplementary file 2 [file Image1.tif]

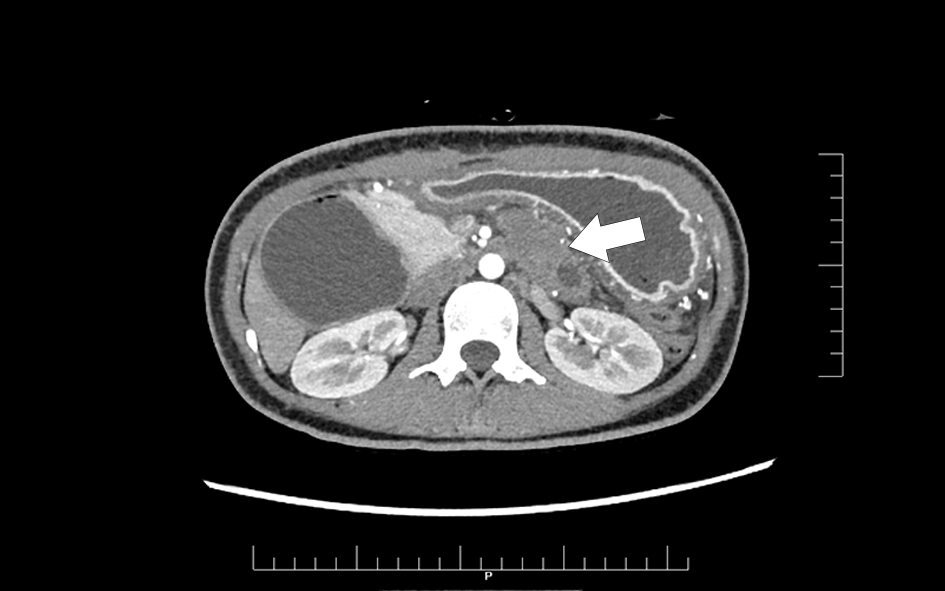

Supplement: Supplementary file 3 [file Image2.tif]

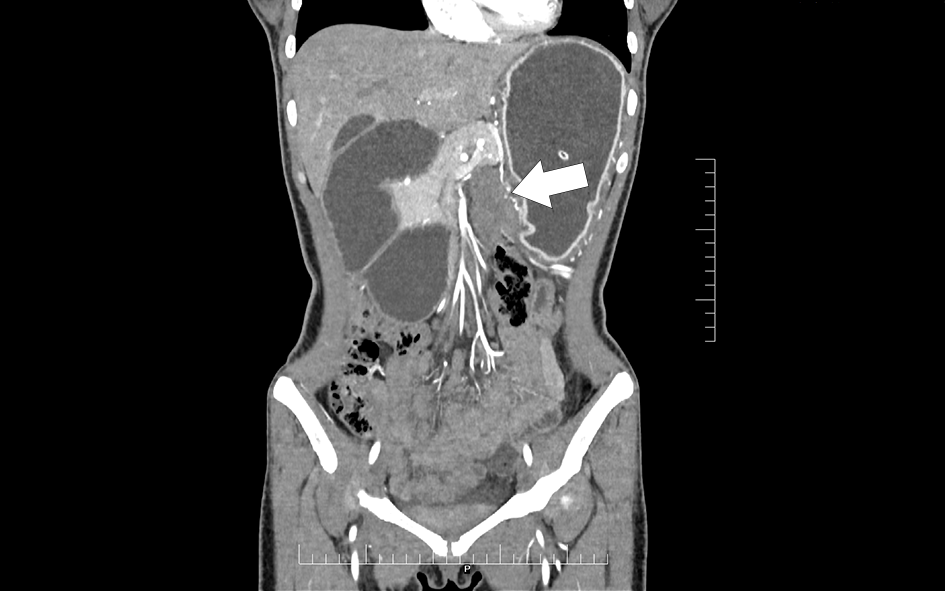

Supplement: Supplementary file 4 [file Image3.tif]

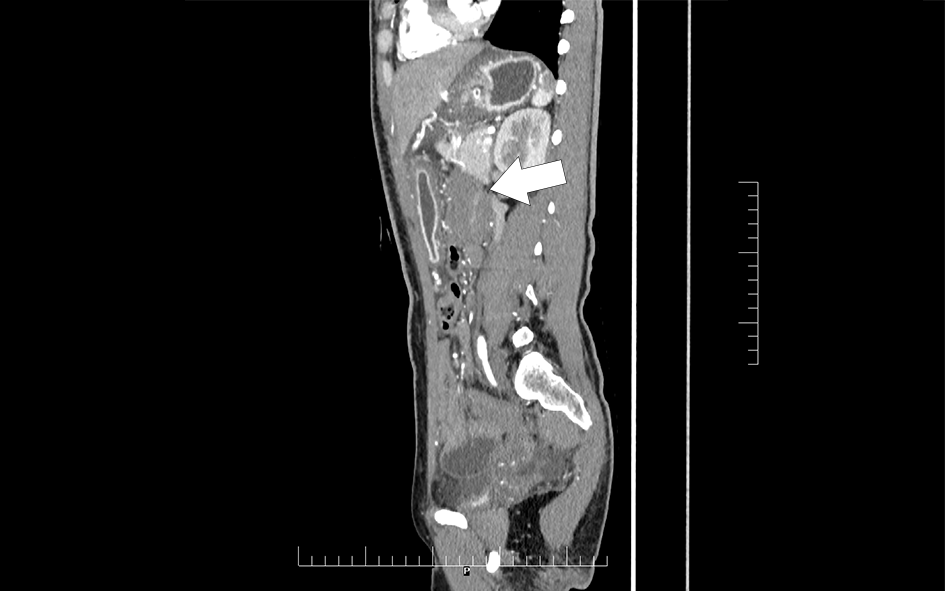

Supplement: Supplementary file 5 [file Image4.tif]

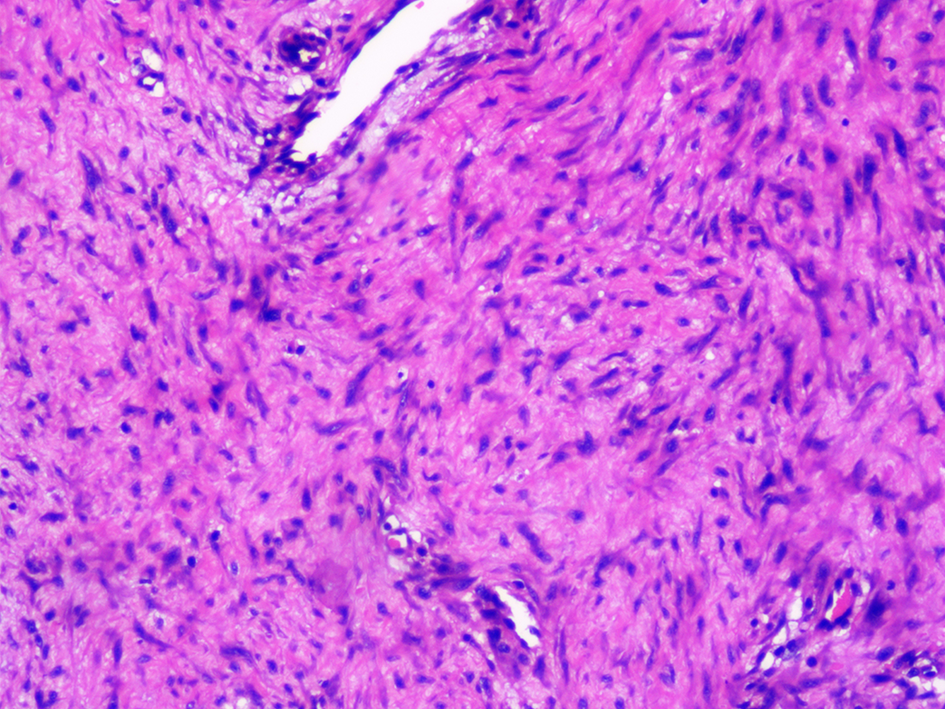

Supplement: Supplementary file 6 [file Image5.tif]

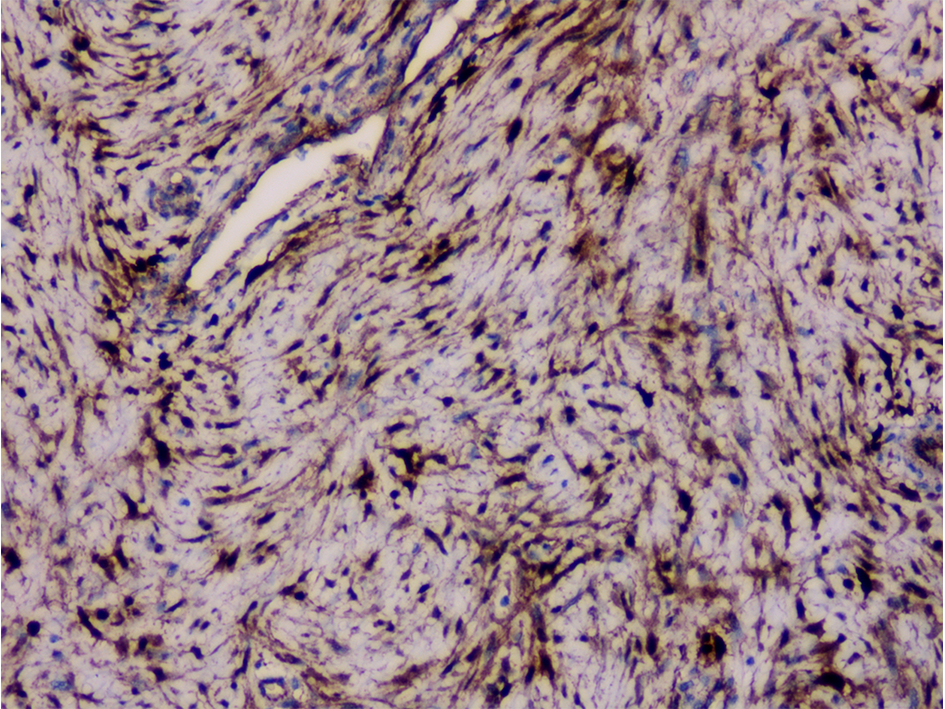

Supplement: Supplementary file 7 [file Image6.tif]

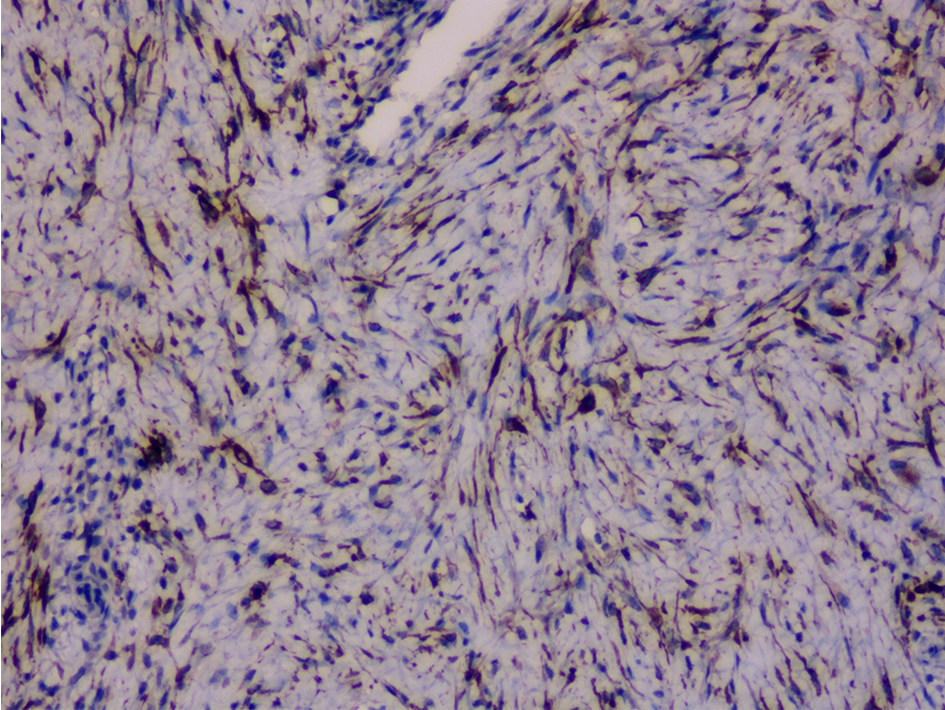

Supplement: Supplementary file 8 [file Image7.tif]

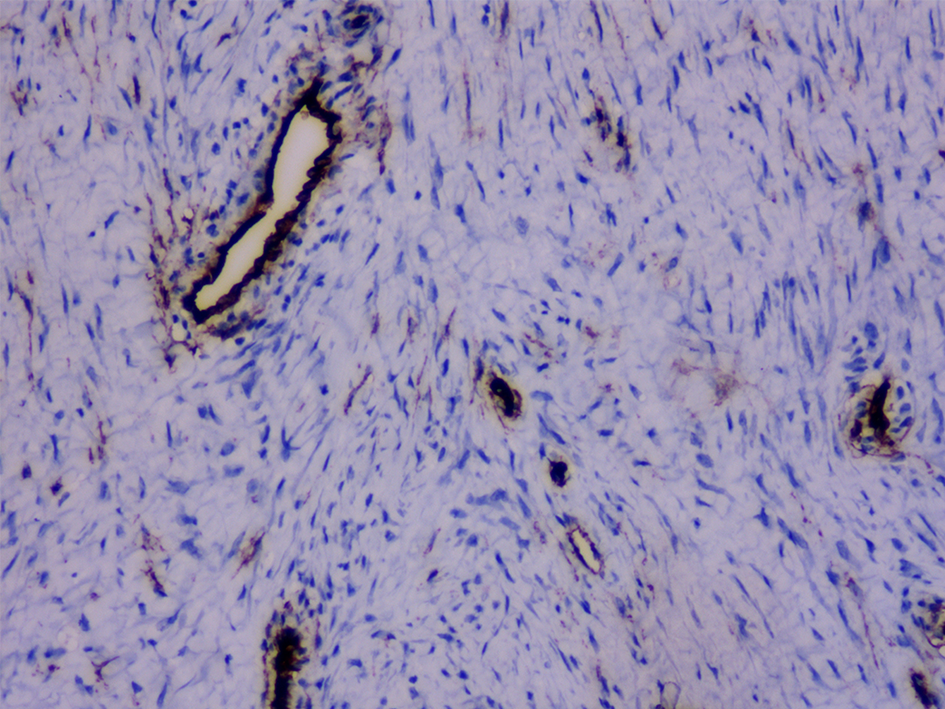

Supplement: Supplementary file 9 [file Image8.tif]

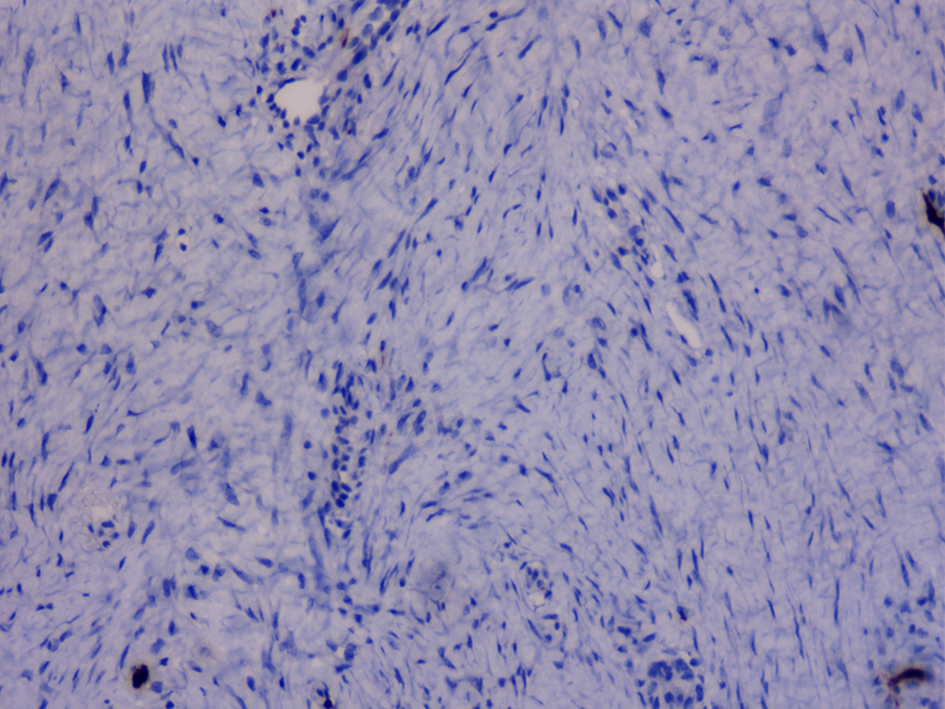

Supplement: Supplementary file 10 [file Image9.tif]

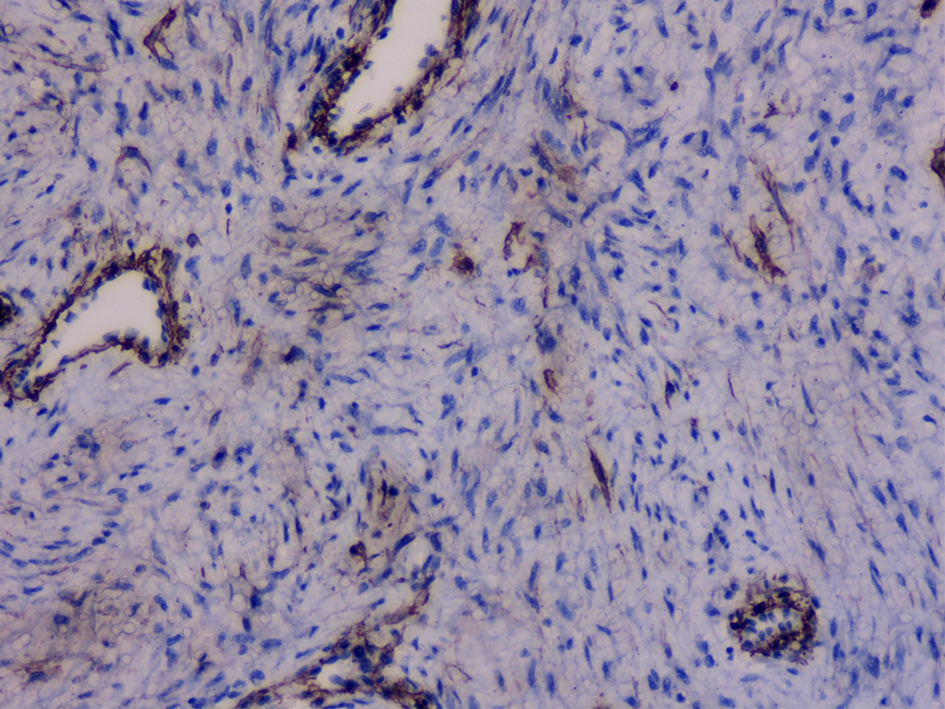

Supplement: Supplementary file 11 [file Image10.tif]
